# Protocol for single-molecule FISH in the developing mouse retinal vasculature

**DOI:** 10.1016/j.xpro.2024.103510

**Published:** 2024-12-12

**Authors:** Josy Augustine, Madeleine R. Smith, Ryan Delaney, Precious O. Owuamalam, Guilherme Costa

**Affiliations:** 1 Wellcome-Wolfson Institute for Experimental Medicine, Queen’s University, Belfast BT9 7BL, UK; 2 Advanced Imaging and Histology Unit, Faculty of Medicine, Health and Life Sciences, Queen’s University, Belfast BT9 7BL, UK

**Keywords:** developmental biology, gene expression, *in situ* hybridization

## Abstract

The developing vasculature of the post-natal mouse retina is a powerful model to discover mechanisms of vessel formation and to test modulators of neovascularization. We present a protocol for single-molecule fluorescent *in situ* hybridization (smFISH) in whole-mount mouse retinas enabling the detection of individual mRNAs in vascular endothelial cells. We describe procedures from initial retina preparation to smFISH and detection. Our approach offers simple steps to overcome challenges related to tissue permeabilization, mRNA and protein co-detection, and post-acquisition image processing.

## Highlights

• Guidance on dissecting neonatal mouse retinas and preparation for smFISH
• Step-by-step protocol for detecting mRNAs in the retinal endothelium
• Steps for mRNA and protein co-detection in the developing retinal vasculature

## Abstract (editor highlights)

Publisher’s note: Undertaking any experimental protocol requires adherence to local institutional guidelines for laboratory safety and ethics.

## Graphical abstract

**Figure:**
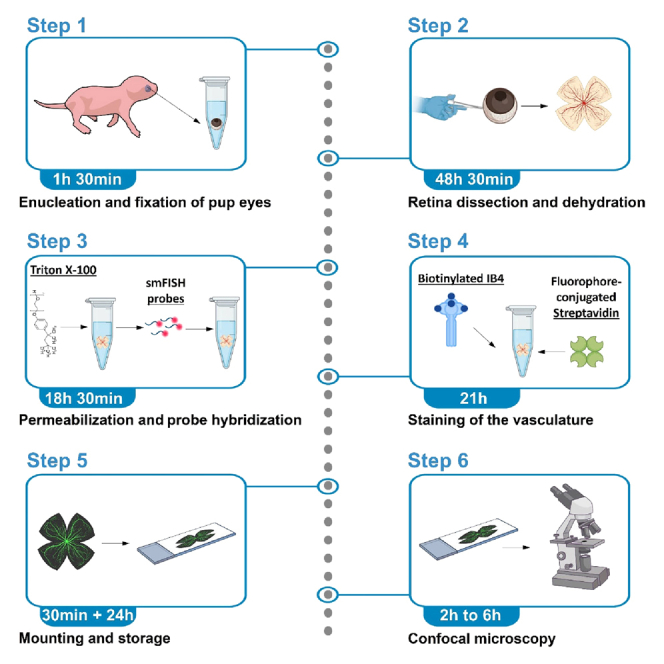

## Before you begin

In contrast to its human counterpart, the development of the mouse retinal vasculature is a post-natal process. In mouse pups, an incipient vascular plexus outgrows from the optic nerve head around birth and gives rise to the superficial vessel layer within the first week (∼P7). These immature vessels undergo extensive rearrangements to mature into a fully functional vessel bed, whilst expanding towards inner layers of the retina, where the deeper (∼P8 to ∼P12) and intermediate (∼P14 to P21) vessel layers are formed.^1^ Due to easy access, this model has been widely used in studies uncovering the expression of genes encoding key modulators of vascular biology. Such discoveries have been aided by RNA *in situ* hybridization (ISH)-based methods, either in whole-mount or sectioned retinas. However, the spatial resolution of the detected RNAs is limited due to signal amplification steps or diffusion of the detection reagents.^2^^,^^3^ By allowing the detection of individual transcripts, as well as precise quantification of their numbers, single-molecule fluorescence ISH (smFISH) is hailed as a transformative addition to the toolkit of RNA detection methodologies. smFISH entails sets of 25 to 50 oligonucleotide probes, each 18 to 22 nucleotides long. The probes are individually labeled with a fluorophore and target distinct regions of the transcripts of interest, allowing their detection with great selectivity and sensitivity.^4^ Despite the successful application of smFISH to the imaging of RNAs in mouse embryonic tissues,^5^ its use in more complex samples such as whole-mount post-natal retinas has remained challenging. Here, we detail a robust protocol for the detection of single mRNAs, with high spatial resolution, in post-natal retinal vasculature. Although this protocol uses two mRNA species as testbeds, it may be employed to detect other transcripts.

### Design smFISH probes

We recommend the use of Stellaris RNA FISH probes from LGC Biosearch Technologies individually labeled with Quasar dyes. Before proceeding with the design of new probes, search for pre-designed sets (https://www.biosearchtech.com/products/rna-fish/designready-stellaris-probe-sets) in the company’s collection, which have been bioinformatically analyzed and experimentally validated for maximum specificity. For custom-designed probes, use the Stellaris RNA FISH Probe Designer (https://www.biosearchtech.com/support/tools/design-software/stellaris-probe-designer) with an input target sequence, to produce a set of up to 48 probes. For reliable detection, a minimum of 25 probes are required. Where the user is struggling to pass this threshold, especially when dealing with short or repetitive targets, the probe spacing can be reduced (from 2 to 1). Increasing the probe length from 18 up to 22 nucleotides and varying the masking level will relax the designer and make more input sequence available for probe design. If the target of choice has non-uniform GC content with distinct GC- and AT-rich regions, it is possible to create a mixed length probe set by combining non-overlapping probes from different design sets (of different probe lengths). Further information regarding probe design and ordering can be found on the LGC Biosearch Technologies website. Once the desired transcript sequence has been filled in, the output containing the probe sequences (up to maximum of 48 probes) is displayed, with probe positions and GC content. To view the position of the probes, there is a drop-down menu with the input sequence and the positions of the probes. After designing the probes, align them against the mouse transcriptome (e.g., NCBI’s BLAST). For hybridization specificity, it is recommended to remove probes with ≥16 nucleotides complementary to non-target transcripts. To optimize this protocol, we used a custom-designed probe set targeting *Pecam1* (Table S1).

### Institutional permissions

All animal maintenance and experiments adhered to the UK Home Office Animals (Scientific Procedures) Act 1986 and were approved by the Animal Welfare Ethical Review Body (AWERB) of Queen’s University Belfast. The experiments also conformed with the Association for Research in Vision and Ophthalmology (ARVO) statement for the use of animals in Ophthalmology and vision research.

### RNase-free handling

**Timing: 30 min**

1. Adhere to RNase-free practices for sample collection, processing, and handling when performing smFISH in post-natal mouse retinas.
  **CRITICAL:** Utilize nuclease-free reagents, filter pipette tips, needles, Petri dishes, and Eppendorf tubes to prevent mRNA degradation at least until the smFISH probe hybridization step is finalized.
  a. Wear a mask and full personal protective equipment when working near or with samples and slides.
  b. Decontaminate workbench and tools using RNase*Zap*, followed by absolute ethanol, and allow them to air dry.
  c. Utilize nuclease-free water for preparing aqueous solutions.

### Preparation of stock and working solutions

**Timing: 1 h**

2. Reconstitute the lyophilized probe stock to 25 μM concentration.
  a. Reconstitute the dried oligonucleotide probe mixture in 200 μL (custom-designed *Pecam1* Quasar 570, 5 nmol) or 40 μL (pre-designed *Gapdh* Quasar 670, 1 nmol) of TE buffer (10 mM Tris-HCl, 1 mM EDTA, pH 8.0) to achieve a stock probe concentration of 25 μM.
  b. Thoroughly mix the solution by pipetting up and down, followed by vortexing and a brief centrifugation.
  c. Aliquot and store the probe stock solution at −20°C.
3. Prepare 20 mL of 10× PBS stock solution.
  a. Add one PBS tablet to a sterile 50 mL Falcon tube.
  b. Pipette 10 mL of nuclease-free water.
  c. Vortex the tube until the solution becomes homogenous.
  d. Complete the volume to 20 mL by adding more nuclease-free water.
4. Prepare 50 mL of 2× PBS working solution.
  a. Pipette 10 mL of 10× PBS solution to a sterile 50 mL Falcon tube.
  b. Add 40 mL of nuclease-free water.
  c. Vortex the tube until the solution becomes homogenous.
5. Prepare 40 mL of 4% formaldehyde in 2× PBS.
  a. Pipette 22 mL of nuclease-free water into a sterile 50 mL Falcon tube.
  b. Add one 10 mL ampule of 16% formaldehyde to the nuclease-free water.
  c. Pipette 8 mL of 10× PBS into the diluted formaldehyde solution and invert the tube to mix thoroughly.

**CRITICAL:** Formaldehyde is toxic and an irritant. Use only within a fume hood to avoid inhalation and direct contact. Always wear gloves and safety glasses when handling it.

6. Prepare 50 mL of PBS working solution.
  a. Pipette 5 mL of 10× PBS solution to a sterile 50 mL Falcon tube.
  b. Add 45 mL of nuclease-free water.
  c. Vortex the tube until the solution becomes homogenous.

## Key resources table

**Table:** 

REAGENT or RESOURCE	SOURCE	IDENTIFIER
**Antibodies**		

Biotinylated isolectin B4 (IB4) (1:150)	Sigma-Aldrich	L2140
Monoclonal Rat CD144 (VE-cadherin) antibody (1:25)	Invitrogen	14-1441-85
Streptavidin Alexa Flour 488 conjugate (1:200)	Invitrogen	S11223
Donkey anti-rat IgG Alexa Fluor Plus 647 (1:150)	Invitrogen	A48272

**Chemicals, peptides, and recombinant proteins**		

Phosphate-buffered saline	Sigma-Aldrich	P4417
Formaldehyde (16%)	Thermo Fisher Scientific	28906
SSC (20×)	Invitrogen	AM9770
Formamide	Sigma-Aldrich	F9037
Triton X-100	Sigma-Aldrich	T8787
Donkey serum	Sigma-Aldrich	D9663
Bovine serum albumin	Sigma-Aldrich	A7906
Dextran sulfate	Sigma-Aldrich	D8906
Nuclease-free water	Invitrogen	AM9932
RNase*Zap*	Invitrogen	AM9780
Blocking reagent (10×)	Roche	11921673001
SuperFrost Plus microscope slides	VWR	631-0108
Surgipath coverslips (22 × 40#1)	Leica Biosystems	3800115G
Prolong Gold antifade reagent	Invitrogen	P36930

**Experimental models: Organisms/strains**		

C57BL/6J, post-natal 3–20 days, either gender	Inotiv	N/A

**Oligonucleotides**		

smFISH probe: custom-designed *Pecam1* Quasar 570, 5 nmol. See Table S1	LGC Biosearch Technologies	SMF-1063-5
smFISH probe: pre-designed *Gapdh* Quasar 670, 1 nmol	LGC Biosearch Technologies	SMF-3140-1

**Software and algorithms**		

Fiji	Schindelin et al.^6^	https://imagej.net/software/fiji/downloads
Imaris	Oxford Instruments	https://imaris.oxinst.com/
RS-FISH	Bahry et al.^7^	https://github.com/PreibischLab/RS-FISH)

**Other**		

Confocal microscope	Leica Microsystems	Stellaris 5
Dissection microscope	Olympus	SZX7
See-saw shaker	Stuart	SSL4
Hybridization oven/shaker	Amersham Biosciences	RPN2511

## Materials and equipment

**Table:** 2× SSC buffer

Reagent	Final concentration	Amount
**20**× **SSC Buffer**	2×	5 mL
**Nuclease-Free water**	N/A	45 mL
Total volume	**N/A**	**50 mL**

Prepare fresh 2× SSC buffer for each round of experiment.

**Table:** Wash buffer

Reagent	Final concentration	Amount
**20**× **SSC Buffer**	2×	2 mL
**Formamide**	10%	2 mL
**Nuclease-Free water**	N/A	16 mL
Total volume	**N/A**	**20 mL**

Prepare fresh formamide wash buffer for each round of experiment.

**CRITICAL:** Formamide is toxic, flammable, and an irritant. Use only within a fume hood to avoid inhalation and direct contact. Always wear gloves and safety glasses when handling it.

**Table:** Hybridization buffer

Reagent	Final concentration	Amount
**Dextran Sulfate**	100 mg/mL	1 g
**20**× **SSC Buffer**	2×	1 mL
**Formamide**	10%	1 mL
**Nuclease-Free water**	N/A	Fill up to 10 mL
Total volume	**N/A**	**10 mL**

Hybridization buffer can be aliquoted and stored at −20°C.

**Table:** IB4 blocking buffer (1×)

Reagent	Final concentration	Amount
**Blocking reagent**	1×	1 mL
**PBS**	N/A	9 mL
Total volume	**N/A**	**10 mL**

Prepare fresh blocking buffer for each round of experiment.

**Table:** Immunofluorescence blocking solution (1×)

Reagent	Final concentration	Amount
**Donkey Serum**	1%	100 μL
**Bovine Serum Albumin**	2%	0.2 g
**PBS**	N/A	Fill up to 10 mL
Total volume	**N/A**	**10 mL**

Prepare fresh immunofluorescence blocking solution for each round of experiment.

## Step-by-step method details

### Enucleation and fixation of post-natal mouse eyes

**Timing: 1 h 30 min**

This step details the enucleation, fixation, and washing of post-natal eyes from mouse pups.

1. Enucleation of post-natal eyes.
  a. Place the humanely euthanized mouse pup on its side.
  b. Using micro spring scissors or 30-gauge needles, carefully remove the skin covering the eyes.
  c. Gently enucleate eyes using microdissection forceps (tweezers) and scissors.
  d. Employ a sterile 30-gauge needle to create an incision in the anterior segment of the eyes.

**CRITICAL:** An ocular incision ensures the fixative permeates the retina and other ocular structures, facilitating effective fixation.

2. Fixation and washing of eyes.
  a. Transfer eyes into individual 2 mL tubes containing 1.8 mL of 4% formaldehyde prepared in 2× PBS for fixation.
    **CRITICAL:** Formaldehyde is a toxic substance. Use only within a fume hood to avoid inhalation and direct contact. Always wear gloves and safety glasses when handling it.
  b. Allow each eye to fix at 21°C for 1 h.
  c. Transfer each eye to cold 2× PBS for 10 min on ice to remove any residual fixative.
    **CRITICAL:** The increased osmolarity of the 2× PBS lowers intraocular pressure, which softens the eyes and aids in subsequent dissection.^2^^,^^8^ It also helps prevent the retinas from curling during dissection.

### Dissection and dehydration of whole-mount retinas

**Timing: 48 h 30 min**

This step describes the preparation and dissection of retinas from the eyes, followed by dehydration in methanol and 70% ethanol (EtOH).

3. Dissection of retinas.
  a. Using tweezers or a modified plastic Pasteur pipette with a widened bore, gently transfer the eyes into a Petri dish containing cold 2× PBS.
    i. Position the dish under a dissecting microscope with a light source.
  b. For each eye, employ a 26-gauge needle or scissors to puncture the corneal periphery.
    i. Cut around the circumference of the cornea and iris and discard the excised tissues.
    ii. Remove the vitreous humor and lens by pulling out gently using tweezers.
  c. Carefully insert tweezers between the retina and sclera, and detach the retina gently from sclera, ensuring the retina remains intact and undamaged.
    ***Note:*** The choroid layer can also be separated gently from the retina. However, if there is a risk of damaging the retinas during this process, it is advisable to leave the choroid layer intact. Retaining the choroid layer should not significantly affect the quality of smFISH and subsequent vessel detection steps.
  d. Rinse the cup-shaped retinal tissue with PBS in a sterile Petri dish.
  e. Segment each retina into lobes by performing 3 to 4 radial incisions reaching approximately two-thirds of the retinal radius using scissors.
    **CRITICAL:** Dividing the retinas into lobes allows flattening of the retinas. Avoid making incisions too close to the optic nerve to prevent the retinas from disintegrating during subsequent processing.
  f. Position the retinas with the retinal vasculature oriented upward and remove excess PBS using a small piece of absorbent paper.
    i. For retinas with lobes that do not spontaneously open completely, gently unfold and flatten them using tweezers.
  g. Slowly pipette cold (−20°C) methanol drop by drop onto the entire retinal surface until the tissue turns white and rigid.
    **CRITICAL:** Introduce methanol gently to prevent curling of the retinas. Methanol is a toxic alcohol. Avoid direct contact with skin, eyes, and clothing. Always wear gloves and safety glasses when handling it.
4. Dehydration of retinas.
  a. Using tweezers or a modified plastic Pasteur pipette with a widened bore, gently transfer each retina to a 2 mL tube containing 1 mL of cold methanol.
  b. Incubate retinas in a freezer (−20°C) for 24 h.
    **Pause point:** Retinas can be stored for up to a week in methanol at −20°C.
  c. After 24 h, wash the retinas twice in 1.8 mL of PBS at 21°C for 5 min each on a shaker at a flat angle to facilitate free horizontal floating of the tissue.
  d. Subsequently, rinse the retinas twice in 1.8 mL of 70% EtOH at 21°C for 5 min each on a shaker at a flat angle.
  e. Incubate the retina in fresh 1.8 mL of 70% EtOH at 4°C for 24 h on a shaker at a flat angle.
    ***Note:*** The dehydration process with methanol and 70% EtOH will aid in fixing the retinas and facilitate permeabilization.

### Permeabilization and smFISH probe hybridization

**Timing: 18 h 30 min**

This step outlines the permeabilization of retinas, followed by the hybridization of smFISH probes.

5. Permeabilization of retinas.
  a. After 24 h, wash the retinas twice in 1.8 mL of 2× SSC at 21°C for 15 min each on a shaker at a flat angle.
  b. Permeabilize the retinas with 500 μL of 10% Triton X (prepared in 2× SSC) for 10 min, with gentle shaking.
    ***Note:*** The duration of permeabilization is optimal for post-natal retinas. However, timing may vary depending on the age of the animals, fixation time, and length of methanol storage.
  c. Terminate the permeabilization by aspirating the solution and wash the retinas twice in 1.8 mL of 2× SSC for 10 min on a shaker at a flat angle.
6. smFISH hybridization.
  a. Incubate the retinas in 500 μL of smFISH wash buffer for 5 min on a shaker.
  b. Add 200 μL smFISH hybridization buffer containing 250 nM Stellaris probe(s) to the retinas and incubate for 16 h at 37°C, with gentle shaking.
    **CRITICAL:** After adding the probe(s) to smFISH hybridization buffer, it is vital to vortex the probe mixture thoroughly for optimal mixing.
    **CRITICAL:** Protect the samples from light from this point onwards.
  c. After probing, wash the retinas twice in 1.8 mL of prewarmed formamide wash buffer at 37°C for 30 min each on a shaker at a flat angle.
    **CRITICAL:** Prewarm the formamide wash buffer to 37°C for at least 30 min.
  d. Wash the retinas twice in 1.8 mL of 2× SSC buffer at 21°C for 5 min each on a shaker at a flat angle.

### Post-smFISH isolectin-B4 staining of the retinal vasculature

**Timing: 21 h**

This step details the washing, blocking and incubations of retinas for labeling with isolectin-B4 (IB4).

7. IB4 staining.
  a. Wash the retinas three times in 1.8 mL of PBS at 21°C for 5 min each on a shaker at a flat angle.
  b. Incubate retinas in 500 μL of IB4 blocking buffer for 1 h, with gentle shaking.
  c. Add 200 μL IB4 blocking buffer containing 1:150 Biotinylated IB4 to the retinas and incubate for 16 h at 4°C, with gentle shaking.
  d. Rinse the retinas four times in 1.8 mL of PBS at 21°C for 15 min each on a shaker at a flat angle.
  e. Add 200 μL IB4 blocking buffer containing 1:200 Streptavidin Alexa Flour 488 conjugate and incubate for 2 h at 21°C, with gentle shaking.
    **CRITICAL:** After adding the Streptavidin conjugate to IB4 blocking buffer thoroughly, it is vital to vortex the probe mixture thoroughly for optimal mixing. Then centrifuge for 20 min at 9800 × *g* (at 21°C) to precipitate any aggregates before adding supernatant to retinas.
  f. Wash the retinas four times in 1.8 mL of PBS at 21°C for 15 min each on a shaker at a flat angle.

### Optional—Post-smFISH immunolabeling and IB4 staining of retinal vasculature

**Timing: 101 h**

This optional step is a replacement for step 7 and describes the washing, blocking, and incubations of retinas for labeling with an antibody of interest and IB4.

8. Immunolabelling and IB4 staining.
  a. Wash the retinas thrice in 1.8 mL of PBS at 21°C for 5 min each on a shaker at a flat angle.
  b. Incubate retinas in 500 μL of immunofluorescence blocking buffer for 2 h at 21°C, with gentle shaking.
  c. Add 200 μL immunofluorescence blocking buffer containing the primary antibody at the desired concentration and 1:150 Biotinylated IB4 to the retinas.
  d. Incubate for 48 h at 4°C, with gentle shaking.
    ***Note:*** We found that this long incubation step results in optimal immunolabeling.
  e. Rinse the retinas four times in 1.8 mL of PBS at 21°C for 15 min each on a shaker at a flat angle.
  f. Add 200 μL immunofluorescence blocking buffer containing secondary fluorophore-conjugated antibody at the desired concentration and 1:200 Streptavidin Alexa Flour 488 conjugate to the retinas.
  g. Incubate for 48 h at 4°C, with gentle shaking.
    ***Note:*** Again, we found that this long incubation step results in optimal immunolabeling.
    **CRITICAL:** After adding the secondary antibody and the Streptavidin conjugate to the immunofluorescence blocking buffer, it is vital to vortex the probe mixture thoroughly for optimal mixing. Then centrifuge for 20 min at 9800 × *g* (at 21°C) to precipitate any aggregates before adding supernatant to retinas.
  h. Wash the retinas four times in 1.8 mL of PBS at 21°C for 15 min each on a shaker at a flat angle.

### Mounting and storage of the retinas

**Timing: 30 min + 24 h**

This step details the mounting and storage of retinas before microscopy.

9. Mounting of the retinas.
  a. Using tweezers or a modified plastic Pasteur pipette with a widened bore, gently transfer the retinas along with a few drops of PBS onto a slide.
    i. Position the slide under a dissecting microscope with a light source.
      **CRITICAL:** Ensure that the retinas remain hydrated at all times during this step and add more PBS if necessary.
  b. Position the retinas with the vasculature oriented upwards.
    i. For retinas with lobes that do not spontaneously open completely, gently unfold and flatten them using tweezers.
  c. Once the retinas are flattened, remove excess PBS using a small piece of absorbent paper.
  d. Dispense 20 μL of Prolong Gold mountant onto a coverslip, aligning it with the position of retinas on the slide.
  e. Invert the coverslip and gently lower it onto the retinas, ensuring the mountant drops correspond with the retinas’ location.
    i. Carefully place the coverslip over the retinas, ensuring no air bubbles are trapped.
  f. Before imaging, incubate the slides with mounted retinas in a slide box for 24 h at 21°C, ensuring that they are flat and protected from light.
    **Pause point:** Samples may be stored at 4°C for up to 2 weeks or at −20°C for extended periods. Thaw frozen slides at 21°C before imaging.

### Imaging of the retinas

**Timing: 2–6 h**

This step outlines the imaging of retinas with a confocal microscope, as it is well-suited for examining thick retinal tissues. The retinas can be imaged in a confocal microscope of choice. We found that a STELLARIS 5 Leica confocal microscope controlled with Leica Application Suite X (LAS X) produces optimal results.

10. Confocal microscopy settings.
  a. Magnification: 100× (1.4 NA HC PL APO CS2 oil).
  b. Resolution: 1024 × 1024 pixels.
  c. Scan speed: 400 Hz.
  d. Z-Step size: 0.2 μm (15–25 stacks).
  e. Line average: 4.
  f. Sensors:
     Laser line 488 nm: PMT 4.
     Laser line 568 nm: PMT 4.
     Laser line 647 nm: PMT 4.
  g. Intensity and gain:
     Laser line 488 nm: 1% and 2.5.
     Laser line 568 nm: 11% and 25.
     Laser line 647 nm: 20% and 10.
    **CRITICAL:** Extended exposure to high-intensity illumination, particularly at high magnifications, should be avoided to prevent photobleaching.
    ***Note:*** We recommend using Fiji^6^ with the RS-FISH^7^ plugin for image processing and spot detection.

### Optional—Deconvolution of confocal images

**Timing: 30 min**

This additional step describes deconvolution of confocal images using LAS X software.

11. Deconvolution settings.
  a. Gold’s Method.
  b. Total iterations = 20.
  c. Refractive index = 1.47.
  d. Select remove background and rescale intensity.

## Expected outcomes

**Figure 1:**
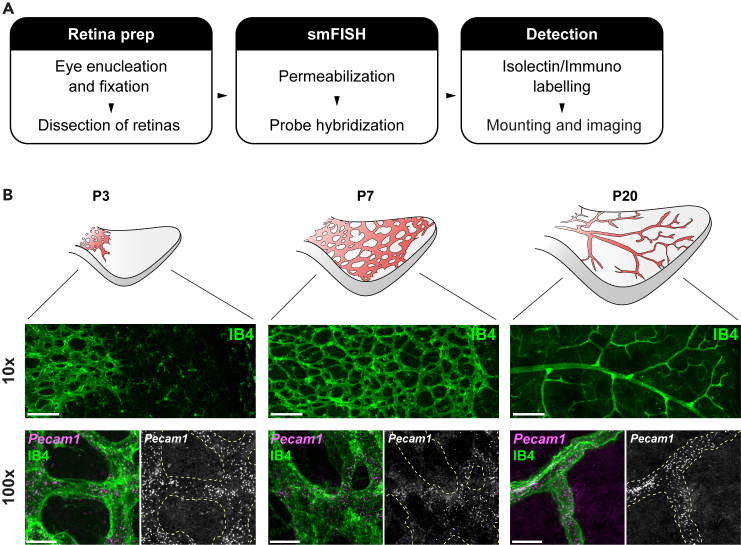
smFISH detects mRNAs in superficial vessels of post-natal mouse retinas (A) Protocol outline highlights key stages and steps. (B) smFISH *Pecam1* spots can be visualized in IB4 labeled vasculature of pup retinas at distinct post-natal stages. Scale bars: 100 μm (10×) and 10 μm (100×).

**Figure 2:**
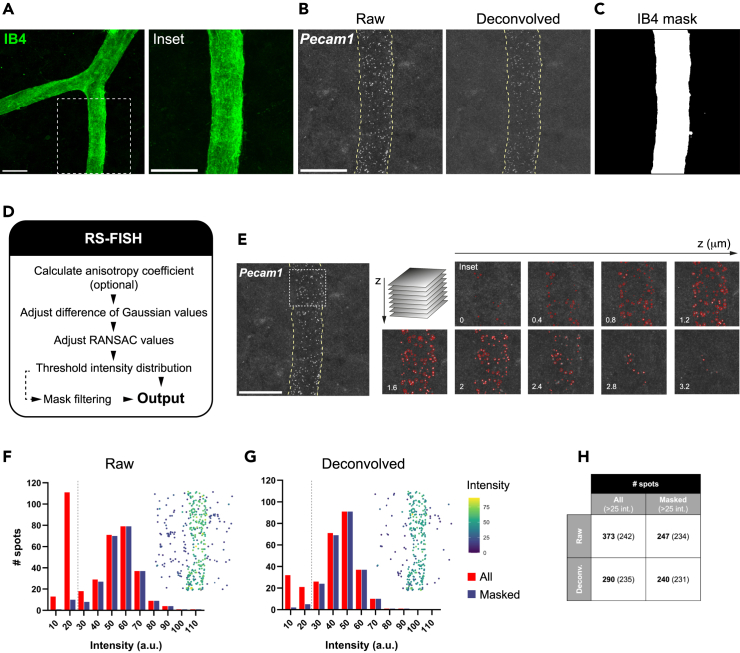
smFISH spot detection in mouse retinal vessels (A) Exemplar of a large P20 retinal vessel labeled by IB4. (B) Maximum projection of a z-stack showing smFISH *Pecam1* raw and deconvolved signal corresponding to the inset region in A. (C) IB4 binary mask generated in Fiji. (D) Main interactive steps of the RS-FISH plugin developed for Fiji. (E) Interactive RS-FISH 3D spot detection overlayed on raw smFISH z-stack and filtered with IB4-based mask; note that changes in circle diameter follow signal intensity across the stack. (F and G) Histograms represent spot counts in (F) raw and (G) deconvolved image; dashed line indicates adjustable threshold applied in RS-FISH to exclude low intensity smFISH signal; point plots overlaying the histograms represent the relative position of all detected spots. (H) Table summarizes spot counts. Scale bars: 20 μm.

The post-natal murine retina is a powerful model for the dissection of mechanisms underpinning physiological and pathological blood vessel formation. Here, we developed an smFISH protocol that allows the unprecedented detection of transcripts with single-molecule resolution in endothelial cells of the growing retinal vasculature (Figure 1A). To aid the identification of vessels during imaging, we add post-smFISH steps of incubation with IB4, classically used to label endothelial cells in mouse tissues. Characteristic diffraction-limited *Pecam1* spots can be visualized in superficial vessels of early (P3), mid (P7) and late (P20) stages of retinal neovascularization (Figure 1B). If necessary, raw confocal microscopy images may be deconvolved to improve signal-to-noise ratio and to clear the background (Figures 2A and 2B). Images with strong IB4 signal may be used to generate threshold-based binary masks for downstream analysis (Figures 2A and 2C). This protocol generates images suitable for downstream imaging processing tools such as RS-FISH, a robust method that uses radial symmetry for spot detection in 3D images developed by the Preibisch lab.^7^ The plugin allows parameter adjustment to optimize spot detection and if necessary, to filter detections within regions of interest based on IB4 masks (Figure 2D). RS-FISH-detected spots are highlighted on the 3D image stacks with circle overlays that change in diameter according to spot intensity across the z-axis (Figure 2E and Video S1). The need for deconvolution may vary with the smFISH probe used and/or image quality and can be inferred using spot detection. In our experience, thresholding spot intensity during RS-FISH processing of raw images results in spot numbers comparable to those detected in the deconvolved counterparts. As expected, most excluded spots are not within the range of the IB4 mask, indicating that these are likely to result from non-specific signals (Figures 2F–2H).

Video S1. Overlay of RS-FISH spot detection on 3D z-stack showing *Pecam1* smFISH, related to Figure 2 and step 9

**Figure 3:**
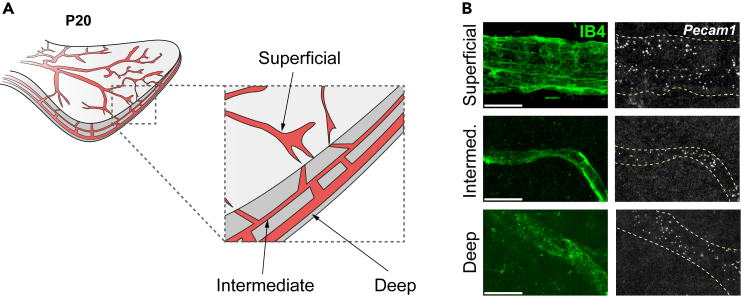
smFISH allows the visualization of transcripts in all three vascular layers of the retina (A) Scheme depicts the superficial, intermediate, and deep vessel layers formed by stage P20. (B) *Pecam1* transcripts can be detected in the 3 vessel plexuses of the P20 pup retina. Scale bars: 10 μm.

**Figure 4:**
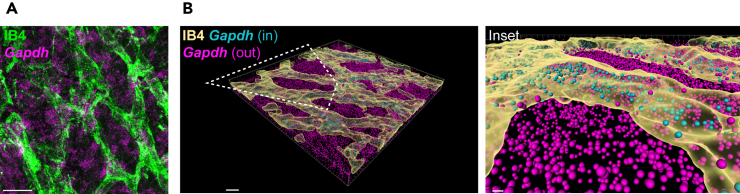
IB4 3D masks permit delineating endothelial expression of transcripts with broad expression patterns (A) smFISH detection of *Gapdh* in a P7 retina labeled with IB4. (B) 3D rendering of retinal vessels delineates *Gapdh* expression in retinal vessels. Scale bars: 20 μm (A), 10 μm (B main) and 2 μm (B inset).

**Figure 5:**
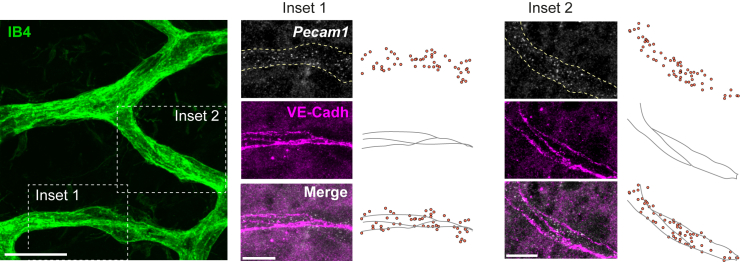
Post-smFISH immunolabeling of retinal vessels Insets of selected regions within P7 retina show smFISH detection of *Pecam1* mRNAs in combination with the endothelial-specific adhesion protein Ve-Cadherin; schemes represent the relative position of RS-FISH detected spots and outlines of Ve-Cadherin immunolabeling. Scale bars: 20 μm (main) and 10 μm (insets).

Our protocol also permits the detection of transcripts in vessels of all three vessel layers of P20 retinas. *Pecam1* spots can be detected in the superficial, intermediate and deep plexuses, indicating that the permeabilization steps of the protocol allow probe access through the retina (Figures 3A and 3B). Furthermore, IB4-based 3D segmentation can be used to delineate endothelial expression domains of mRNAs with ubiquitous expression (Figure 4, Video S2). Lastly, optional post-smFISH immunolabeling is possible. For example, *Pecam1* transcripts can be co-detected with Ve-Cadherin, an endothelial-specific adhesion molecule present in cell-cell junctions (Figure 5).

Video S2. 3D rendering of z-stack showing *Gapdh* smFISH and IB4 labeling, related to Figure 4 and step 9

## Limitations

We developed a protocol for smFISH in whole-mount retinas with high resolution. The protocol will be of particular relevance for studies exploring endothelial gene expression in expanding vessel beds. Of note, the approach we present is limited to a couple of exemplar transcripts that we selected as testbeds. Whether other transcripts can be equally detectable via smFISH is yet to be determined. Furthermore, the protocol does not provide a strategy that generates data suitable for endothelial cell segmentation, which can be challenging in whole-mount retinas. Thus, some further optimization is required for accurate transcript quantification at single-cell level and mapping of subcellular mRNA localization. Lastly, the presented protocol is limited to the detection of mRNAs within vascular endothelial cells, which can be easily labeled with IB4. Although we anticipate that the protocol may be used as a springboard for the development of further RNA detection protocols in non-endothelial retinal cells, the applicability of post-smFISH labeling tactics that facilitate the identification of other retinal cell types needs to be tested.

## Troubleshooting

### Problem 1

Retina whole mounts are damaged during manipulation.

### Potential solution

Correct eye fixation (Step 2) is critical for the successful maintenance of tissue integrity throughout the successive incubation and wash steps. However, over-fixation may result in poor smFISH signal. We suggest 1 h incubation with 4% Formaldehyde for the post-natal stages tested in this protocol, but the fixation timing may be adjusted. Try short increments to the Formaldehyde incubation time if the whole-mounts are too soft and become damaged by the end of the protocol.

### Problem 2

smFISH produces poor signals, hindering mRNA detection.

### Potential solution

If smFISH spot intensity is low, we suggest optimizing tissue permeabilization (Step 5) and probe concentration (Step 6).

• Optimal permeabilization of the retina whole-mounts is a determinant factor for smFISH probe penetration. We found that incubating the retinas with 10% Triton X-100 for 10 min produces good results when post-smFISH immunolabeling is necessary. However, increasing the incubation time may help permeabilizing later stage retinas to produce stronger signals in harder, deeper regions of the tissue.
• When post-smFISH immunofluorescence is not necessary, we found that permeabilizing the whole-mounts with Proteinase K (10 μg/mL) for 5–10 min instead of Triton-X produces excellent smFISH results with minimal tissue damage and no detrimental effects on IB4 signal.

### Problem 3

Immunolabeling generates poor signals, hindering protein co-detection.

### Potential solution

Post-smFISH immunolabeling may be useful for researchers interested in RNA and protein co-detection (Optional Step 7). However, we noticed that primary antibodies that commonly produce a strong signal when used in immunolabeling-only protocols can generate worse results when used in combination with smFISH. In this scenario, we recommend increasing the primary antibody concentration in a step-wise manner until achieving a desirable signal-to-noise ratio. If using an untested antibody, we suggest following an immunofluorescence protocol for retina whole-mounts (e.g., Tual-Chalot et al.^8^) to determine antibody concentration and accurate expression pattern of the target protein. Antibody concentration in post-smFISH approaches can then be optimized from this stage onwards.

## Resource availability

### Lead contact

Further information and requests for resources and reagents should be directed to and will be fulfilled by the lead contact, Guilherme Costa (g.costa@qub.ac.uk).

### Technical contact

Technical questions on executing this protocol should be directed to and will be answered by the technical contact, Josy Augustine (j.augustine@qub.ac.uk).

### Materials availability

No new materials were generated in this study.

### Data and code availability

No datasets were generated or analyzed during this study.

## Acknowledgments

We are grateful to Professor Tim M. Curtis for critical feedback and sharing reagents and materials. We also thank members of the Biological Services Unit and Advanced Imaging and Histology Unit at Queen’s University Belfast for their technical support. This work was supported by the Medical Research Council (MR/X001164/1 to G.C.) and Wellcome Trust (225339/Z/22/Z to G.C.). Parts of the graphical abstract for this article were created using BioRender.com.

## Author contributions

J.A. developed the methodology, performed the experiments, and wrote the manuscript. M.R.S. performed the experiments. R.D. performed formal analysis. P.O.O. wrote and reviewed the manuscript. G.C. supervised the project, performed formal analysis, prepared the visualization, and wrote the manuscript.

## Declaration of interests

The authors declare no competing interests.
