# Supplementary material for: Protocol for single-molecule FISH in the developing mouse retinal vasculature
Source: STAR Protoc. 2024 Dec 12;5(4):103510. doi: 10.1016/j.xpro.2024.103510 (PMC11699415; doi:10.1016/j.xpro.2024.103510)
Supplement: Document S1. Table S1 [file mmc1.pdf]

Supplemental information

Table S1: Oligonucleotide sequences of smFISH Stellaris® probe targeting *Pecam1*, related to Step 6.

Oligonucleotide	Sequence (5'-3')	Oligonucleotide	Sequence (5'-3')
*Pecam1*_1	agacttctgcagactgaagc	*Pecam1*_25	gcttatctgtgaatgttgct
*Pecam1*_2	tccatatggatgctgttgat	*Pecam1*_26	gggtgggaatggcaattatc
*Pecam1*_3	tcgcatcgtccttatagaac	*Pecam1*_27	atcacaacttcatccactgg
*Pecam1*_4	ctgaggaatgacgtagctct	*Pecam1*_28	tttcacagagcaccgaagta
*Pecam1*_5	catcactgtgcatttgtact	*Pecam1*_29	tttgttgtgccaaaacgctt
*Pecam1*_6	tcaatcgtggtttttcctt	*Pecam1*_30	gaggctgtacagtagtactg
*Pecam1*_7	ggaacaattgaccgtcacga	*Pecam1*_31	ggcaaggaagactctgactg
*Pecam1*_8	tcgcttgacaaactttgtcc	*Pecam1*_32	atgacaaccaccgcaatgag
*Pecam1*_9	cgttggaggtcttatctatc	*Pecam1*_33	gcatttggctgcaactatta
*Pecam1*_10	aatggggaattccatgagca	*Pecam1*_34	ttggagttcagaagtggagc
*Pecam1*_11	aacactaacacgtggtcctg	*Pecam1*_35	ccacactaggctcagaaatc
*Pecam1*_12	gcaatttgaatccggacagg	*Pecam1*_36	gttttactgcatcatttcca
*Pecam1*_13	caggggggcttgatttcaaac	*Pecam1*_37	ttccacttctgtgtattcta
*Pecam1*_14	tgtcacttgaactatgcacc	*Pecam1*_38	aatacgtgcacaggactctc
*Pecam1*_15	ctgtaaactcctggaccaag	*Pecam1*_39	gaaggattactgctttcggt
*Pecam1*_16	tacaatcgccttgtcttttt	*Pecam1*_40	aggcagcggggtttaaaatt
*Pecam1*_17	actgagtagacagcttcact	*Pecam1*_41	tctctgtatacccaacatga
*Pecam1*_18	gatacggtttgattccactt	*Pecam1*_42	atattccagggcattattct
*Pecam1*_19	gttatgttgaccatgatgct	*Pecam1*_43	agacacaacatttcagcctc
*Pecam1*_20	gaactctaacttcggcttgg	*Pecam1*_44	cacggagaagtactctgtct
*Pecam1*_21	cggcgatcttgctgaaattc	*Pecam1*_45	tgaggtctgtcttcaagtga
*Pecam1*_22	aaatactgggcttcgagagc	*Pecam1*_46	aaggaagatcaaggtgcgct
*Pecam1*_23	atctcagacttggcatcatg	*Pecam1*_47	cagaccttaggaaaccgttt
*Pecam1*_24	agagtctggaagtcactctt	*Pecam1*_48	acacaaaatgtcggcagctc
